# Specific barrier response profiles after experimentally induced skin irritation in vivo

**DOI:** 10.1111/cod.12981

**Published:** 2018-04-02

**Authors:** Maryam Soltanipoor, Tasja Stilla, Christoph Riethmüller, Jacob P. Thyssen, Judith K. Sluiter, Thomas Rustemeyer, Tobias W. Fischer, Sanja Kezic, Irena Angelova‐Fischer

**Affiliations:** ^1^ Coronel Institute of Occupational Health, Academic Medical Centre, Amsterdam Public Health Research Institute University of Amsterdam Amsterdam The Netherlands; ^2^ Department of Dermatology VU University Medical Centre Amsterdam The Netherlands; ^3^ Department of Dermatology University of Lübeck Lübeck Germany; ^4^ Centre for Nanotechnology, Serend‐ip GmbH, Centre for Nanotechnology Münster Germany; ^5^ National Allergy Research Centre, Department of Dermatology and Allergy Copenhagen University Hospital Herlev‐Gentofte, University of Copenhagen Hellerup Denmark

**Keywords:** biomarkers, irritant contact dermatitis, skin barrier

## Abstract

**Background:**

Recently, natural moisturizing factors (NMFs) and corneocyte surface topography were suggested as biomarkers for irritant dermatitis.

**Objectives:**

To investigate how exposure to different irritants influences corneocyte surface topography, NMF levels and the barrier function of human skin in vivo.

**Methods:**

Eight healthy adult volunteers were exposed to aqueous solutions of 60% n‐propanol, 0.5% sodium lauryl sulfate (SLS), 0.15% sodium hydroxide, and 2.0% acetic acid, and distilled water, in a repeated irritation test over a period of 96 hours. Erythema, transepidermal water loss (TEWL), skin hydration, the dermal texture index (DTI) and NMF levels were measured at baseline, and after 24 and 96 hours.

**Results:**

SLS and sodium hydroxide had the most pronounced effects on erythema and TEWL. Although n‐propanol caused only slight changes in TEWL and erythema, it showed pronounced effects on skin hydration, NMF levels, and the DTI. NMF was the only parameter that was significantly altered by all investigated irritants. The changes in the DTI were inversely associated with NMF levels and skin hydration.

**Conclusion:**

Skin barrier impairment and the inflammatory response are irritant‐specific, emphasizing the need for a multiparametric approach to the study of skin irritation. NMF levels seem to be the most sensitive parameter in detecting irritant‐induced skin barrier alterations.

## 1 INTRODUCTION

Irritant contact dermatitis (ICD) is a common inflammatory skin disease that can be elicited by single or repeated exposure to chemicals with irritating properties, such as detergents, organic solvents, alkaline agents, and acids. The pathomechanism of ICD remains incompletely understood; however, impairment of the skin barrier function plays a central role, triggering homeostatic and innate immune responses.^1^, ^2^ Recently, Koppes et al^3^ reported 2 novel biomarkers of irritant skin reactions to sodium lauryl sulfate (SLS): the dermal texture index (DTI) as a measure of the corneocyte topography, and the levels of natural moisturizing factors (NMFs). As shown by the use of atomic force microscopy (AFM), SLS dramatically increased the number of circular nano‐objects protruding above the corneocyte surface, as shown by the DTI,^3^ and decreased NMF levels at the same time. In contrast to the SLS‐induced increase in the DTI, no changes in the DTI were observed in skin affected by allergic contact dermatitis.^3^ These observations suggest that the alterations in the corneocyte surface topography might be attributed to the skin barrier damage caused by the irritant, rather than to inflammation itself.^4^ Therefore, we were interested in whether the changes in the DTI and NMF levels were SLS‐specific or also occurred with other skin irritants. Furthermore, we wanted to investigate the relationships of the DTI and NMF levels with skin bioengineering parameters, including erythema, transepidermal water loss (TEWL), and skin hydration. In the present study, we investigated the early and late changes in the morphological, biochemical and bioengineering parameters following repeated exposure to different classes of water‐soluble skin irritants in the human skin in vivo.

## 2 METHODS

### 2.1 Study population

Healthy adult volunteers aged 20 to 65 years (*n* = 8; 3 females and 5 males; median age 24.5 years) without a history of skin or systemic diseases were recruited for the study. The exclusion criteria comprised intensive ultraviolet (UV) exposure in the test area within the last 6 weeks before inclusion and/or during the study, pregnancy, or lactation. The participants were not allowed to use skin care products in the test area (back) within 24 hours prior to the baseline measurements and for the entire duration of the study (5 days). The protocol was approved by the Ethics Committee of the University of Lübeck (No. 14‐111), and all participants gave written informed consent beforehand.

### 2.2 Irritants and mode of exposure

Exposure to the irritants and distilled water as a control was performed under occlusion with large Finn Chambers (12 mm in diameter; Smart Practice, Barsbüttel, Germany). Two rows, each comprising 4 test (irritant‐exposed) and 2 control (water and non‐exposed, normal skin) fields, were marked left and right on the skin of the upper‐middle back. Fifty microlitres of aqueous solutions of the following irritants were applied: 0.5% SLS (99.0% purity; Sigma‐Aldrich, Steinheim, Germany), 0.15% sodium hydroxide (NaOH) (Mallinckrodt Baker, Deventer, The Netherlands), 60.0% n‐propanol (1‐propanol) (Merck, Darmstadt, Germany), and 2.0% acetic acid (AcA) (Roth, Karlsruhe, Germany). The test chambers were applied twice daily for 30 minutes, within an interval of 3 to 4 hours, on 4 consecutive days by use of a previously validated and published protocol.^5^ The fields of the first row were examined at baseline and 24 hours later, and those of the second row were examined at baseline and 96 hours later.

### 2.3 Bioengineering parameters: erythema, TEWL, and capacitance

All measurements were performed at baseline and 24 and 96 hours later under controlled ambient conditions (temperature 21°C ± 1°C, 40%‐50% relative humidity). The skin irritant response was measured by non‐invasive assessment of erythema, TEWL, and skin hydration. Erythema was measured with the Skin‐Colorimeter CL400 and expressed in the L*a*b*system; TEWL was measured with the open chamber system (Tewameter TM300), and skin hydration was assessed by measuring capacitance (Corneometer CM825); all devices were from Courage and Khazaka Electronics (Cologne, Germany). For each parameter, two consecutive measurements per field were performed by the same trained observer and according to published guidelines.^6^, ^7^, ^8^, ^9^

### 2.4 Biochemical parameters: NMF levels

NMF levels were determined in stratum corneum (SC) tape strips collected with 14‐mm D‐Squame discs (CuDerm, Dallas, Texas). Tape stripping was performed on day (D) 2 and on D5, respectively, 24 and 96 hours after the first application of the irritants, as previously described.^10^ Six consecutive tapes from each field were obtained and stored in sterile 1.5‐mL Eppendorf tubes (Eppendorf, Hamburg, Germany) at −80°C until analysis; the fifth tape was used for analysis. The NMF components (histidine, 2‐pyrrolidone‐5‐carboxylic acid, *trans‐*urocanic acid, and *cis‐*urocanic acid) on the tape were extracted with 400 μL of 25% (wt/wt) ammonia solution, evaporated to dryness, and reconstituted in 200 μL of pure water. The extracts were diluted 1:1 with mobile phase prior to high‐performance liquid chromatography with UV detection analysis. To compensate for variable amounts of the SC on the tape, the amount of SC was estimated from the protein levels determined in the aqueous extract after ammonia extraction, as previously described.^11^ Briefly, the amount of protein was determined from an aliquot of 75 μL of the extract by use of a Pierce Micro BCA protein assay kit according to the procedure of the manufacturer (Thermo Fisher Scientific, Rockford, Illinois; hereafter referred to as the Pierce assay). Bovine serum albumin was used as a standard for preparation of a calibration curve. The NMF concentration was normalized for the protein amount on the tape, and expressed as mmol NMF/g protein.

### 2.5 Morphological parameters: corneocyte surface topography

The corneocyte surface morphology was analysed by AFM, as described previously.^12^ Briefly, the third consecutive tape strip was subjected to AFM measurements carried out with a Multimode atomic force microscope equipped with a Nanoscope III controller and software version 5.30 sr3 (Digital Instruments, Santa Barbara, California). Silicon nitride tips on V‐shaped gold‐coated cantilevers were used (0.01 N/m, MLCT; Veeco, Mannheim, Germany). Imaging was performed at ambient temperature with forces of <1 nN at 1 to 3 scan lines per second (1‐3 Hz) with a resolution of 512 × 512 pixels. For texture analysis, subcellular scan areas of 20 × 20 μm^2^ were recorded. For a larger overview, images of 70 × 70 μm^2^ were recorded. Topographical data of the corneocyte surfaces were analysed with the nAnostic method, by the use of custom‐built, proprietary algorithms (Serend‐ip, Münster, Germany) evaluating each nanostructure protruding from the mean surface level, referred to as circular nanosize object (CNO). The CNOs were automatically filtered according to their size and shape; only structures of positive local deviational volume <500 nm in height and with an area of <1 μm^2^ were considered. The DTI represents the number of these features for an area of 20 × 20 μm^2^ of cell surface per image.^13^

### 2.6 Statistical analyses

Statistical analyses were performed with graphpad prism 7.0 (Graphpad Software, La Jolla, California). A *P* value of <.05 was considered to be statistically significant. Data distribution was assessed with the Shapiro‐Wilk test. The differences in the bioengineering parameters (erythema a* value, capacitance, and TEWL) between baseline and the respective readings at 24 and 96 hours were compared by the use of a two‐sided paired Student *t* test. To test the effect of water, differences between the water control (occlusion) and the non‐exposed skin site at 24 and 96 hours were analysed with a two‐sided paired Student *t* test. In order to assess the neat effect of the irritant, the differences between the values of the bioengineering parameters measured at 24 and 96 hours and the corresponding baseline values were calculated for each irritant (Δ values) and compared with the Δ values of the water control by the use of repeated measures ANOVA followed by Dunnett's multiple comparisons test. The correlation between the investigated parameters was evaluated with Pearson's correlation analysis (normal distribution) or with Spearman's rank correlation test (skewed distribution). In the tables and figures, the data are presented as mean values ± standard error of the mean (SEM), unless otherwise indicated.

## 3 RESULTS

### 3.1 Bioengineering parameters

**Figure 1:**
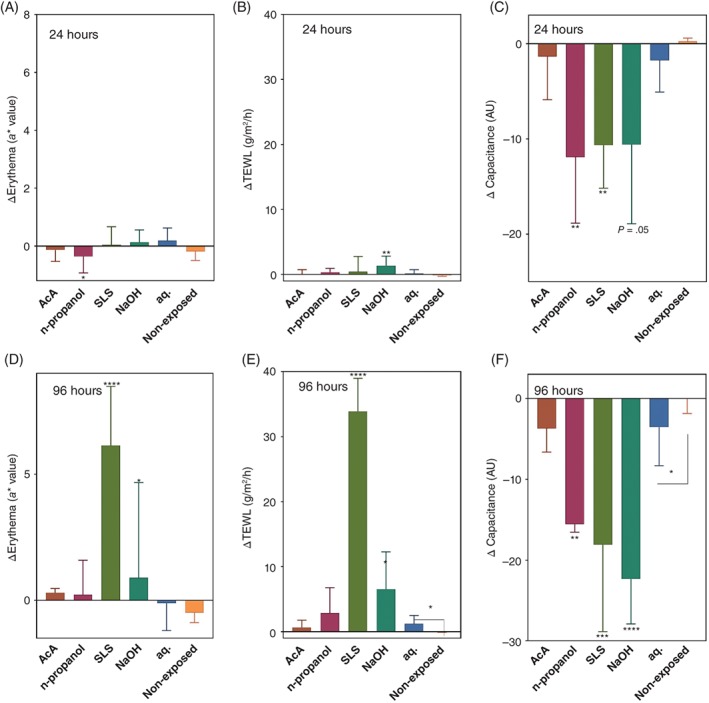
Comparison of the changes in the bioengineering parameters at 24 and 96 hours from baseline (Δ values). **(A),** Erythema, 24 hours. **(B),** Transepidermal water loss (TEWL), 24 hours. **(C),** Capacitance, 24 hours. **(D),** Erythema, 96 hours. **(E),** TEWL, 96 hours. **(F),** Capacitance, 96 hours. Results are shown as mean value ± standard error of the mean. Test fields: acetic acid (AcA), n‐propanol, sodium lauryl sulfate (SLS), sodium hydroxide (NaOH), occlusion with water (aq.), and non‐exposed skin site. The Δ values were compared with those of the water‐exposed skin site by the use of repeated measures ANOVA followed by Dunnett's multiple comparisons test. The difference between the water‐exposed site (aq.) and the non‐exposed skin site was compared by use of a Student *t* test. *P < .05, **P < .01, ***P < .001, ****P < .0001. AU, arbitrary units

The values of erythema (a* value), TEWL and capacitance at baseline and 24 and 96 hours later are shown in Table 1. At baseline, there were no significant differences in any of the measured parameters between the test and control fields (data not shown). Of the studied irritants, SLS exerted the most pronounced effect, and after 96 hours there were significant differences from the baseline values for all bioengineering parameters (Table 1). The water‐exposed skin site showed a significant increase in TEWL and a decrease in capacitance after 96 hours as compared with the corresponding baseline values. Furthermore, the water‐occluded skin site showed a significantly higher TEWL and a lower capacitance at 96 hours than non‐exposed skin (Figure 1).

**Table 1:** Erythema (*a* * value), transepidermal water loss (TEWL) and capacitance at baseline and 24 and 96 hours after repeated exposure to acetic acid (AcA), n‐propanol, sodium lauryl sulfate (SLS), sodium hydroxide (NaOH), and distilled water (aq.) (*N* = 8)

	a* values, mean ± SEM				TEWL (g/m^2^/h), mean ± SEM				Capacitance (AU), mean ± SEM			
	Baseline	24 h	Baseline	96 h	Baseline	24 h	Baseline	96 h	Baseline	24 h	Baseline	96 h
AcA	10.0 ± 0.7	9.8 ± 0.6	10.3 ± 0.7	10.6 ± 0.6	5.5 ± 0.2	5.7 ± 0.2	5.5 ± 0.2	6.3 ± 0.4*	36.5 ± 2.5	34.0 ± 3.5	32.2 ± 2.5	32.1 ± 1.5*
n‐propanol	9.8 ± 0.7	9.5 ± 0.7	9.8 ± 0.7	10.3 ± 0.7	5.3 ± 0.2	6.2 ± 0.7	5.4 ± 0.2	9.4 ± 1.1*	37.8 ± 3.3	25.3 ± 3.5***	37.7 ± 3.3	23.1 ± 4.3****
SLS	9.8 ± 0.8	10.0 ± 0.8	9.9 ± 0.6	16.0 ± 0.9***	5.6 ± 0.2	6.7 ± 0.5	5.6 ± 0.2	37.1 ± 3.5****	36.8 ± 2.6	27.1 ± 3.7**	37.1 ± 2.5	18.1 ± 4.4***
NaOH	9.9 ± 0.7	10.2 ± 0.7	10.0 ± 0.7	12.1 ± 1.2	5.6 ± 0.2	7.3 ± 0.5*	5.5 ± 0.2	13.2 ± 1.6**	35.3 ± 2.1	22.2 ± 1.7**	35.3 ± 2.1	11.3 ± 1.3****
aq.	9.6 ± 0.6	9.7 ± 0.6	9.9 ± 0.6	9.4 ± 0.6	5.6 ± 0.2	5.7 ± 0.3	5.6 ± 0.2	6.8 ± 0.3*	36.1 ± 2.0	34.0 ± 2.9	36.0 ± 2.0	31.1 ± 2.4*
Non‐exposed	9.4 ± 0.6	9.2 ± 0.6	9.7 ± 0.6	9.0 ± 0.7	5.4 ± 0.2	5.4 ± 0.2	5.5 ± 0.1	5.5 ± 0.2	36.6 ± 2.1	37.0 ± 2.1	36.4 ± 2.1	37.0 ± 3.1

AU, arbitrary units; SEM, standard error of the mean.

* *P* < .05,

** *P* < .01,

*** *P* < .001 and

**** *P* < .0001

as compared with baseline. Asterisks refer to the significance level for the difference between the values measured at 24 and 96 hours and their corresponding baseline values.

As all investigated irritants were applied in water, which apparently affected the skin barrier, the effect of the irritants was assessed by comparing the changes from the baseline values (Δ values) at 24 and 96 hours with the respective Δ values of the water control. The Δ values for erythema (a* value), capacitance and TEWL are shown in Figure 1. At 24 hours, only n‐propanol induced a significant difference in the Δ values of erythema, and NaOH induced a significant difference in TEWL. In contrast, n‐propanol and SLS induced a significant change from baseline for capacitance, and NaOH showed a tendency to decrease capacitance (*P* = .05). At 96 hours, repeated exposure to SLS and NaOH resulted in significant differences in the Δ values for all measured parameters (Figure 1 D, E, F). At the same time point, AcA exposure did not induce significant differences in any of the outcome parameters, and n‐propanol induced a significant difference (ie, decrease) only with regard to capacitance.

### 3.2 Biochemical and morphological parameters: NMF and DTI

**Figure 2:**
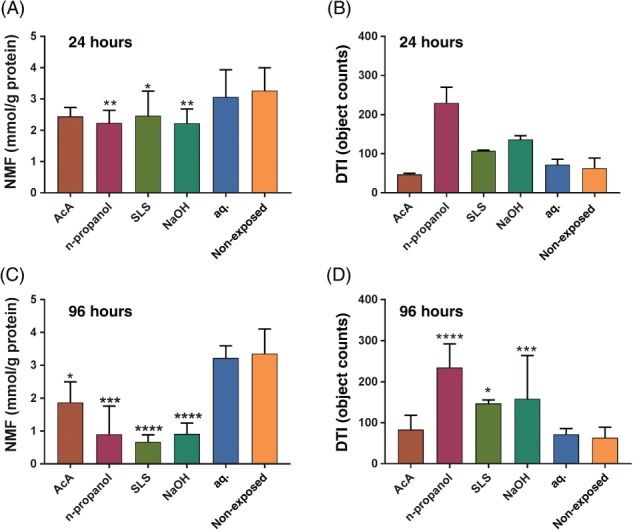
Natural moisturizing factors (NMFs) and dermal texture index (DTI) measured on the stratum corneum tapes collected at 24 and 96 hours after initial exposure. **(A),** NMF, 24 hours. **(B),** DTI, 24 hours. **(C),** NMF, 96 hours. **(D),** DTI, 96 hours. Test fields: acetic acid (AcA), n‐propanol, sodium lauryl sulfate (SLS), sodium hydroxide (NaOH), distilled water (aq.), and non‐exposed skin. The values for the irritants were compared with those for the water‐exposed skin site by the use of repeated measures ANOVA followed by Dunnett's multiple comparisons test (mean ± standard error of the mean). *P < .05, **P < .01, ***P < .001, ****P < .0001

The values of NMF and DTI, measured in the SC tapes collected at 24 and 96 hours after the first irritant exposure, are shown in Figure 2. Repeated application of SLS, NaOH and n‐propanol led to significantly lower NMF levels than the corresponding water control as early as 24 hours after initial exposure (Figure 2A,B). After 96 hours, there were reductions from the baseline value of 22% for AcA, 55% for n‐propanol, 75% for SLS, and 65% for NaOH. Furthermore, 96 hours after the first application of the test chambers, the NMF levels of the water‐exposed skin site were significantly lower than those of the non‐exposed site.

**Figure 3:**
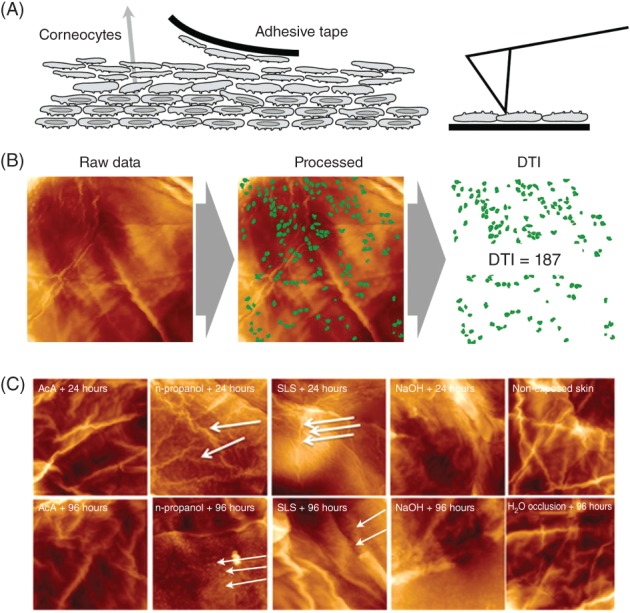
Nanostructure of human corneocytes after irritant exposure in vivo. **(A),** Corneocytes were obtained with the tape stripping method. The basal (bottom) faces of corneocytes adhering to the tape were imaged by the use of atomic force microscopy (AFM). **(B),** 20 μm scans were analysed by computer vision using proprietary algorithms to count circular structures (dermal texture index [DTI]); the presented DTI values are the mean of 10 images. **(C),** Representative images of the corneocyte surface, assessed on the third consecutive tape strip after irritant exposure. Irritant exposures: acetic acid (AcA), n‐propanol, sodium lauryl sulfate (SLS), sodium hydroxide (NaOH), water occlusion, and non‐exposed (normal) skin. Thick white arrows indicate elongated fibres (upper panel), and thin white arrows indicate circular nano‐objects

Representative images of the corneocyte surface topography of the irritant‐exposed and control fields captured by AFM at, respectively, 24 and 96 hours after initial exposure are shown in Figure 3. Already after 24 hours (Figure 3C, upper panel), thinning of fibres, wrinkles and elongated spots were observed on the n‐propanol‐exposed and SLS‐exposed sites. After 96 hours, characteristic circular nano‐objects appeared after exposure to n‐propanol, SLS, and NaOH (Figure 3C, lower panel). The AFM images of the AcA‐exposed site were similar to those of the control skin sites, and also in agreement with their DTI values. The changes in topography observed after n‐propanol, SLS and NaOH exposure were consistent with the elevated DTI (number of circular nano‐objects per surface area), as shown in Figure 2B,D. Note that the AFM measurements after 24 hours were performed in only 2 subjects. Although statistical evaluation of these data was not meaningful, the increasing trend for the DTI suggests that the changes in surface topography occurred early.

**Figure 4:**
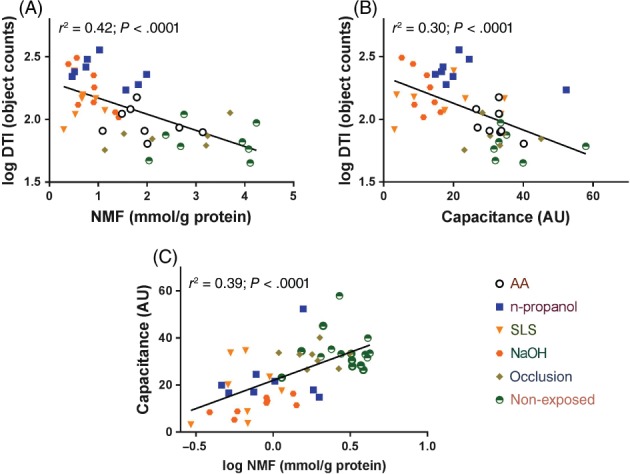
The relationship between the dermal texture index (DTI), and natural moisturizing factor (NMF) levels (**A**), and skin hydration (capacitance) (**B**), measured after 96 hours. Correlation between the capacitance and NMF (**C**) *r* ^2^: determination coefficient of the linear regression analysis. AA, acetic acid; AU, arbitrary units; SLS, sodium lauryl sulfate

To examine the associations between the measured parameters, a correlation analysis was performed. The results of the correlation analysis are shown in Table S1. Figure 4 shows the linear regression lines with corresponding coefficients of determination (*r* ^2^) for the relationship between the DTI (log values) and the parameters relevant for skin hydration: NMF levels and capacitance. For the regression analysis, data for all irritants measured at 96 hours after initiation of exposure were included. As shown in Figure 4, there were inverse correlations between the DTI and, respectively, NMF levels and capacitance. Furthermore, the NMF level was positively associated with capacitance.

## 4 DISCUSSION

In the present study, we investigated and compared the effects of repeated exposure to different classes of water‐soluble irritants on functional, biochemical and morphological parameters of the skin barrier in vivo. Exposure to the skin irritants led to an increase in the DTI, reflecting changes in the corneocyte topography. The DTI was inversely correlated with the NMF levels, which showed decreases after exposure to all irritants. Regarding the bioengineering parameters, the results showed a clear irritant‐specific effect, which has been shown previously.^5^, ^10^, ^17^, ^24^ These irritant‐specific responses may be explained by different mechanisms underlying the skin barrier damage, dependent on the intrinsic nature of the irritant,^7^, ^14^ and suggest that the parameter of choice to assess irritation should largely depend on the type of irritant applied. Whereas erythema has traditionally been the most commonly used parameter to visually assess the irritating properties of a chemical, in the present investigation we did not find significant changes from baseline after exposure to AcA and n‐propanol after 96 hours. This might, at least partly, be explained by their weak irritant capacity, which has been shown previously.^15^, ^16^, ^17^ However, after exposure to n‐propanol, skin capacitance, which is commonly used as a measure of skin hydration, was strongly reduced as early as 24 hours after the first exposure. Skin hydration is of ultimate importance for the skin barrier function, as it influences not only skin plasticity, but also the activities of various proteases involved in desquamation, lipid synthesis, and inflammatory responses.^18^ The water content of the skin is largely dependent on the levels of hygroscopic NMF within the corneocytes,^19^ and also on the SC lipid organization and size of the individual corneocytes that regulate TEWL. The strong and rapid decreases in NMF levels and skin hydration, with TEWL remaining unchanged, suggest that the effect of n‐propanol on skin hydration is probably caused by a decrease in NMF levels rather than by its interference with the lipid bilayers. In addition to its marked effect on NMF levels, n‐propanol also caused significant changes in corneocyte surface topography, resulting in an increase in the number of the circular nano‐objects, expressed as the DTI. In contrast to the effects exerted by n‐propanol predominantly on skin hydration and the DTI, exposure to SLS and NaOH caused significant changes in all measured biophysical parameters (erythema, TEWL, and capacitance), as well as in NMF levels and the DTI. This might be explained by multiple mechanisms by which these alkaline irritants affect the skin barrier.^20^ Literature data show that SLS can affect both protein and lipid structures of the SC. For example, SLS interacts with the SC proteins, resulting in transient swelling of corneocytes.^21^ After an initial increase in hydration, the water‐holding capacity of the SC subsequently decreases, and this, in turn, will result in skin dryness.^22^ In addition to its effect on NMF levels, SLS induces changes in the lipid lamellae organization,^23^ which is consistent with the significant increase in TEWL, as also found in the present study.

The pronounced decrease in skin hydration found after exposure to the irritants can at least partly be explained by the marked decrease in NMF levels, ranging from 22% to 75% from the baseline value, with the most marked effect being induced by SLS and the least marked effect being induced by AcA, which is in agreement with previous studies using the same irritant concentrations and mode of exposure.^10^, ^24^, ^25^ Among all investigated parameters, the NMF level was shown to be the most sensitive with regard to irritant damage, as it was significantly decreased by all studied irritants. For example, for AcA, the NMF level was the only parameter that differed with respect to the corresponding water control. Moreover, the NMF level was the only parameter that was able to detect changes between the non‐exposed skin and water‐occluded skin sites. The precise mechanisms for NMF reduction remain so far unknown, but possible pathways include irritant‐induced effects on the proteolysis of filaggrin to NMF components, reduced expression of filaggrin, or leakage of NMF from the corneocytes because of damage of the cornified and/or lipid envelope. For example, both SLS and NaOH are alkaline substances that cause elevated skin pH and might decrease the activity of the enzymes involved in filaggrin proteolysis.^26^, ^27^

However, Koppes et al observed increased activity of two key enzymes involved in filaggrin proteolysis, bleomycin hydrolase and calpain‐1, after exposure to SLS in the human skin in vivo.^3^ On the other hand, SLS is known to denature proteins of the cornified envelope and increase its permeability,^28^ which may lead to the leakage of NMF components from the corneocytes.^27^ A similar mechanism is also likely to explain the effects of a strong corrosive agent with an alkaline pH, such as NaOH.^14^, ^29^ Further evidence that the reduction in NMF levels may be caused by skin barrier damage is provided by the finding of increased plasmin activity after exposure to SLS.^3^ Elevated plasmin activity has previously been associated with a damaged skin barrier.^3^ Also, n‐propanol is known to induce changes in the SC; for example, it has pronounced denaturing effects on the SC proteins, profilaggrin processing, and desquamatory SC enzymes,^30^ which is consistent with marked decreases in skin capacitance and NMF levels, and changes in surface topography. A recent study showed the presence of another aliphatic alcohol, 2‐propanol, between the solid keratin rods inside the corneocytes, which might explain the morphological changes induced by n‐propanol in the present study.^31^ Our hypothesis that reductions in NMF levels might be associated with skin barrier damage is consistent with the significant inverse correlation of NMF levels with the DTI as a measure of the corneocyte surface topography. All irritants, except for AcA, induced a significant increase in the DTI, and changes such as thinning of fibres, wrinkles and elongated spots were observed as early as 24 hours after initial exposure to n‐propanol and SLS. After 96 hours, characteristic circular nano‐objects appeared, most abundantly after exposure to n‐propanol and SLS.

The DTI has recently been suggested as a parameter that might be used to distinguish allergic from irritant contact dermatitis,^3^ on the basis of findings that SLS induced a strong increase in the DTI, whereas, in the same study, no changes in the DTI after exposure to contact allergens were observed. Here, we extend these findings by showing that the effect on the DTI can also be induced by unrelated irritants with different physicochemical properties. The nature of the DTI has not yet been clarified, but data from recent studies in atopic and contact dermatitis patients^13^ have consistently shown that the DTI is inversely associated with NMF levels, as is also confirmed by the results of our present study. The finding that the DTI was increased in SLS‐exposed skin, but not in experimentally induced allergic contact dermatitis,^3^ suggests that inflammation as such is unlikely to be the underlying cause of the observed changes in the corneocyte surface topography. These observations are in agreement with the findings of the present study, which showed stronger correlations of the DTI with NMF and capacitance than with erythema as 1 of the main signs of inflammation. It may be speculated that the changes in the corneocyte surface texture are caused by decreased skin hydration resulting from reduced NMF levels and/or increased TEWL. This is consistent with the published literature reporting similar changes in the morphology of the corneocyte surface in diseases associated with skin dryness, such as diabetes,^32^ psoriasis, and ichthyosis vulgaris.^33^ Reductions in NMF levels resulting from exposure to skin irritants or genetic factors such as filaggrin gene loss‐of‐function mutations in ichthyosis would lead to decreased hydration and shrinkage of the corneocytes, which might, in turn, cause the observed changes in the cell surface texture.

The present study has several limitations. The study had an explorative character and the sample size was small, partly because the preceding power calculation was based on the previous data on SLS, which is a potent irritant with strong effects on NMF levels and the DTI. Larger‐scale studies are ongoing. Furthermore, the application of the irritants at only 1 concentration does not allow conclusions to be drawn regarding to what extent the observed effects on the measured parameters were dose‐dependent. Nevertheless, our results show significant differences in the barrier response to common water‐soluble irritants, and substantiate the need for the use of a multiparametric approach based on functional, biochemical and morphological parameters to assess skin irritancy in vivo.

### Conflict of interest

The PhD project of MS is funded by DEB Group Ltd. The sponsor was not involved in study design, study execution, or writing of the article. The authors declare no potential conflict of interests.

## Supporting information


**Table S1.** Spearman correlation coefficients for the relationship between investigated parameters measured at 24 and 96 hours.
