# Supplementary material for: Specific barrier response profiles after experimentally induced skin irritation in vivo
Source: Contact Dermatitis. 2018 Apr 2;79(2):59–66. doi: 10.1111/cod.12981 (PMC6099430; doi:10.1111/cod.12981)
Supplement: Supplementary file 1 — Table S1. Spearman correlation coefficients for the relationship between investigated parameters measured at 24 and 96 hours. [file COD-79-59-s001.doc]

Supplementary Table 1. Spearman correlation coefficients for the relationship between investigated parameters measured at 24 and 96 h.

| **24 h** | Erythema (a*values) | Capacitance | TEWL | DTI | NMF |
| --- | --- | --- | --- | --- | --- |
| Erythema (a*values) |  | -0.207 | -0.256 | -0.071 | -0.009 |
| Capacitance | -0.207 |  | -0.098 | -0.252 | 0.507^****^ |
| TEWL | 0.256 | -0.098 |  | 0.024 | -0.228 |
| DTI | -0.071 | -0.252 | 0.024 |  | -0.738^*^ |
| NMF | -0.009 | 0.507^****^ | -0.228 | -0.738^*^ |  |

| **96 h** | Erythema (a*values) | Capacitance | TEWL | DTI | NMF |
| --- | --- | --- | --- | --- | --- |
| Erythema (a*values) |  | -0.470^***^ | 0.546^****^ | 0.345^**^ | 0.543^****^ |
| Capacitance | -0.470^***^ |  | 0.617^****^ | 0.594^****^ | 0.596^****^ |
| TEWL | 0.546^****^ | -0.617^****^ |  | 0.542^****^ | 0.792^****^ |
| DTI | 0.345^**^ | -0.594^****^ | 0.542^****^ |  | 0.672^****^ |
| NMF | 0.543^****^ | 0.596^****^ | 0.792^****^ | 0.672^****^ |  |

Level of significance: *p* <0.05*; *p* < 0.01 **; *p* < 0.001 ***; *p* < 0.0001; *****p* < 0.00001
